# Visceral Leishmaniasis Relapse in HIV Patients—A Role for Myeloid-Derived Suppressor Cells?

**DOI:** 10.1371/journal.pntd.0003132

**Published:** 2014-09-11

**Authors:** Rafael Van den Bergh, Jo A. Van Ginderachter, Elio Schouppe, Belete A. Desimmie, Asrat Hailu, Patrick De Baetselier, Johan van Griensven

**Affiliations:** 1 Laboratory for Cellular and Molecular Immunology, Vrije Universiteit Brussel, Brussels, Belgium; 2 Myeloid Cell Immunology Laboratory, VIB, Brussels, Belgium; 3 Operational Research Unit, Medical Department, Médecins Sans Frontières – Operational Centre Brussels, Brussels, Belgium; 4 Department of Pharmaceutical and Pharmacological Sciences, Katholieke Universiteit Leuven, Leuven, Belgium; 5 Faculty of Medicine, Addis Ababa University, Addis Ababa, Ethiopia; 6 Department of Clinical Sciences, Institute of Tropical Medicine, Antwerp, Belgium; National Institute of Allergy and Infectious Diseases, United States of America

## Background

Visceral leishmaniasis (VL) is a disseminated protozoan infection caused by the *Leishmania donovani* spp. complex and is transmitted by phlebotomine sand flies. Globally, over 200 million people are at risk of contracting VL, and when left untreated, the disease is universally lethal. The human immunodeficiency virus (HIV) pandemic has been one of the main driving forces behind the increased spread of VL over the last 20 years [1]. Sub-Saharan Africa is at the epicentre of this detrimental synergy: in East Africa, the most intense HIV-VL interplay takes place, with HIV prevalence amongst VL cases ranging from 15% to more than 40% in certain areas of Ethiopia and South Sudan. In this region, the highly virulent *L. donovani* prevails, and a large proportion of VL patients present in advanced stages of HIV infection.

Comorbidities of HIV and other infectious diseases such as tuberculosis, cryptococcosis, or viral hepatitis manifest a number of general characteristics including accelerated disease progression, higher rates of adverse outcomes, and therapy-associated complications like the immune reconstitution inflammatory syndrome (IRIS) [2]. While HIV-VL coinfection is also associated with accelerated HIV and VL progression and a poor prognosis, it appears to be governed by a number of unique and poorly explained features. One of these unique features is that even after virological suppression under antiretroviral therapy (ART), patients often remain clinically and immunologically in a state of immunodeficiency and anergy, with diffuse organ spread of parasites [1], [3]. Such patients are characterised by high rates of anti-VL therapy failure, which can be either primary failure or recurring parasitological relapses. In other patients, the unique entity of “active chronic visceral leishmaniasis” was described, entailing continuous asymptomatic parasite replication under therapy, interspersed with symptomatic secondary VL episodes [4]. The observed therapeutic failure contrasts sharply with, e.g., the IRIS events observed in other coinfections, which—while detrimental—are indicative of a partial and possibly even over-exuberant restoration of pathogen-specific immune responses [2]. This therapeutic failure in HIV-VL poses a major challenge to programmes facing a high burden of the coinfection—however, it remains poorly understood and under-researched.

## VL Relapse in HIV-VL Patients: Answered and Unanswered Questions

A recent meta-analysis identified a number of clinical risk factors for VL relapse in HIV-VL patients, including previous VL episodes, low baseline CD4+ T cell counts, an absence of CD4+ T cell increase at follow-up, and an absence of secondary prophylaxis [5]. At the mechanistic level, the precise processes underpinning VL relapse remain ill-characterised: so far, this has only been examined in animal models of coinfection [6], [7]. However, circumstantial evidence suggests that, in the host-pathogen dyad, parasite-related factors are less likely to contribute to therapeutic failure, as modulation of drug susceptibility of parasites in HIV-VL coinfection was either not observed or could be adequately explained by differences in past drug exposure or transmission route [8], [9]. Reinfection of HIV patients, rather than true VL relapse, is likewise not strongly supported by the existing evidence [10]. On the other hand, some evidence has been garnered for the remaining explanation—an aberrant immune response [5], [7].

Since successful anti-VL therapy is known to require a protective Th1 immune response, it is not unexpected to observe VL relapses in severely immunocompromised HIV patients. However, HIV patients on successful ART undergo relatively rapid functional immune reconstitution and should—in similarity with the IRIS-associated immune responses observed in other coinfections—be able to mount a response capable of clearing the parasite during anti-VL treatment early after therapy initiation, irrespective of the stage of HIV infection. The failure of coinfected patients to control VL under ART and VL treatment thus alludes to a persistent immunodeficiency or “dampened” Th1 response as a consequence of VL in these patients [11]. This may also be reflected in the observed tolerance to VL parasites in coinfected patients, where immune responses to the parasite are dampened despite high tissue parasite loads. To better understand this persistence, we must consider the mechanisms by which VL manages to suppress the immune system.

## A New Player in the Field

Immune suppression during VL appears to hinge on the production of IL-10 (reviewed in [12]). Various immune suppressive cell types have been implicated in VL-associated immune suppression, with varying degrees of evidence. The role of regulatory T lymphocytes (Tregs) has been assessed in multiple models (reviewed in [13])—a contribution of Tregs to persistent immune suppression under ART cannot be ruled out, particularly as several studies have documented a skewed reconstitution of Tregs in the early phases of ART, which could lead to a relative dominance of the suppressive cell population. However, it seems unlikely that Tregs alone would be capable of mediating long-term persistent immune suppression in HIV-VL coinfection, as the Treg/T effector ratio tends to normalise following the first few months of ART and, particularly, as the functional contribution of Tregs to VL is still questionable (reviewed in [12]). Other cell types that have been implicated in VL-associated immune suppression include dendritic cells, mediating T lymphocyte suppression through IL-10-dependent and -independent pathways [14], and myeloid-derived suppressor cells (MDSC) [15]–[18].

Here, we propose that MDSC may contribute at least in part to the persistent immune suppression underlying therapeutic failure in HIV-VL patients. This little-known cell type represents a heterogeneous population of immature myeloid cells with marked antigen-specific immunosuppressive capacities, elicited by (partial) inhibition of myeloid cell maturation. MDSC were first identified in models of tumour immune evasion (reviewed in [19]), but their functional contribution to other models of immune evasion, such as during parasitic infections, is increasingly recognised [15], [18]. Specifically, in the context of leishmaniasis, a systemic expansion of MDSC was observed in animal models primed with leishmania parasites [16], [17]. As such, they could contribute at least partially to VL-associated immune suppression, in a similar fashion as observed during tumour immune evasion, potentially perturbing both T lymphocyte proliferation and functionality [15]. Interaction with leishmania-specific T lymphocytes could even convert putative MDSC to non-specific suppressor cells, further exacerbating the HIV-VL-associated immune dysfunction [20].

During VL in otherwise immunocompetent individuals, expansion of MDSC populations could be an attempt at keeping the detrimental excessive immune response to VL and overall systemic immune activation [21] in check. Furthermore, they may even contribute directly to parasite control through production of nitric oxide [22]. However, in severely immunocompromised individuals such as HIV-VL coinfected patients, the presence of a dominant MDSC population—which, as a myeloid cell population, would not suffer HIV-mediated depletion on the scale of the lymphocyte population—could effectively prevent the immune system from coming fully online under ART. MDSC-mediated antigen-specific immune suppression, while relatively innocuous and possibly even beneficial in immunocompetent individuals, could represent an insurmountable obstacle for the reconstitution of the VL-specific immune response.

## Testing the Hypothesis

The main barriers to testing this hypothesis are of a logistical nature: HIV-VL coinfections are prevalent in sufficient numbers only in resource-poor settings, where advanced functional immunological analysis is challenging and establishing clinical cohorts is arduous. However, if a dedicated research initiative could overcome these challenges, the hypothesis could be tested in a relatively straightforward study design. Surface markers for MDSC have been described, both in murine and, more recently, in human models of disease, and quantification of (types of) MDSC in peripheral blood samples of HIV-VL patients could be performed through flow cytometry [23]. A longitudinal three-arm study, consisting of HIV+VL+, HIV+VL−, and HIV−VL+ patients would therefore suffice to provide a proof-of-principle for the putative presence of MDSC during HIV-VL coinfection. The different groups should be carefully matched on key factors such as ART regimen, baseline CD4 cell count, and HIV-1 viral load. For VL cases, analysis of peripheral blood could possibly be complemented with analysis of diagnostic tissue aspirates. Cell isolation, suppression studies, and potentially intervention trials using MDSC targeting [24] could then be introduced in a second phase to provide more conclusive evidence of a central role of MDSC in HIV-VL-associated immune suppression.

Identification of physiologically relevant MDSC populations in HIV-VL coinfected patients as putative contributors to VL therapy failure would pave the way for a host of follow-up studies. Such studies could, on the one hand, include research into improved management of coinfected patients in programmes confronted with a high HIV-VL burden, using simple drugs known to either deplete MDSC or inhibit their suppressive activity, such as vitamin A derivatives, COX2 inhibitors, and ROS inhibitors [19], [24]. On the other hand, laboratory studies on novel methods of averting MDSC-mediated immune suppression of VL in coinfections or into the possibility of controlling MDSC through, e.g., the iNOS/arginase balance [19] can be developed. In addition, a putative role for MDSC in an HIV coinfection model would represent a unique patho-immunological phenomenon and would be a major addition to the existing insights into the fundamentals of infectious diseases.
